# Occupational Physical Activity and Cardiometabolic Risk Factors: A Cross-Sectional Study

**DOI:** 10.3390/nu15061421

**Published:** 2023-03-15

**Authors:** Montserrat Gómez-Recasens, Silvana Alfaro-Barrio, Lucia Tarro, Elisabet Llauradó, Rosa Solà

**Affiliations:** 1 Medical Service of Fomento de Construcciones y Contratas, Delegación Catalunya II, 43007 Tarragona, Spain; montserrat.gomez@urv.cat (M.G.-R.); salfarob@fcc.es (S.A.-B.); 2 Facultat de Medicina i Ciències de la Salut, Functional Nutrition, Oxidation, and Cardiovascular Diseases Group (NFOC-Salut), Health Education and Promotion, and Healthy Environment Chair, Universitat Rovira i Virgili, 43201 Reus, Spain; rosa.sola@urv.cat; 3 Institut d’Investigació Sanitària Pere Virgili (IISPV), 43204, Reus, Spain; 4 Hospital Universitari Sant Joan de Reus, IISPV, Universitat Rovira i Virgili, 43204 Reus, Spain

**Keywords:** occupational physical activity, cardiometabolic risk factors, workplace, cross-sectional

## Abstract

Contradictory data exist on the impact of occupational physical activity (OPA) on cardiovascular health. We aimed to evaluate the association between OPA and cardiometabolic risk factors. A cross-sectional study was performed in an environmental services company in 2017 (Spain). OPA was classified by work categories as being low (≤3 METs) or moderate−high (>3 METs). Multiple linear and logistic binary regression models were used to assess the associations between OPA and cardiometabolic risk factors related to obesity, blood pressure, blood lipids, and associated medical conditions, adjusted by age, sex, alcohol consumption, and global physical activity. In total, 751 employees were included (547 males and 204 females), and 55.5% (*n* = 417) had moderate−high OPA. Significant inverse associations were observed between OPA and weight, body mass index, waist circumference, waist−hip ratio, and total cholesterol both overall and in males. OPA was significantly inversely related to dyslipidemia overall and in both sexes, while the overweight plus obesity rate was inversely related only in the total and male populations. OPA was associated with a better cardiometabolic risk factor profile, particularly in males. The fact that our models were also adjusted by global physical activity highlights the associations obtained as being independent of leisure time physical activity effects.

## 1. Introduction

The workplace has been identified as a suitable environment to promote healthy life-styles for chronic disease prevention [1] due to the large population involved and the increasing trend in working hours/day [2,3]. Workplace environment contributes to the vitality of workers in order to prolong their working life. Health promotion programs in the workplace have been shown to have a positive impact on workers’ physical and mental health [4,5]. Currently, there is an increase in sedentary lifestyles caused by a mechanization of work, technology, etc., in the workplace, and by changes in the environment and society outside workplace [6]. Sedentary lifestyles are associated with worse health and an increase in early morbidity and mortality. It is known that leisure-time physical activity (LTPA), the physical activity (PA) performed in free time, decreases the risk of chronic diseases such as cardiovascular disease (CVD), obesity, diabetes mellitus (DM), hypertension, and some types of cancer [7,8]. The role of occupational PA (OPA), performed at work on health, however, still needs to be defined, independent of the LTPA performed.

Studies focused on OPA and CVD risk have reported di-verse results. In some studies, a beneficial effect of OPA on cardiovascular and total mortality has been described [9,10]. One study in patients with diabetes concluded that not only LTPA, but also occupational and commuting PAs, are important components of a healthy lifestyle among patients with diabetes [9]. Another study in non-risk cardiovascular individuals concluded that the lack of LTPA and a sedentary occupation are associated with an increased risk of ischemic heart disease death [10]. Data from the Danish Nurse Cohort Study showed that a low influence at work (defined as the level of influence an individual normally has on the organization of their daily work) was the key factor for increased risk of ischemic heart disease in nurses exposed to strenuous OPA [11]. In some studies, low OPA has been shown to be poorly or not associated with health status [12], and meta-analyses data showed no interrelationship between PA at work and CVD [13]. Data from the Copenhagen City Study concluded that a higher LTPA is associated with reduced adverse cardiovascular events and all-cause mortality risk, while a higher OPA is associated with increased risks, independent of each other [14]. Previous data in the frame of this study have shown that high OPA was associated with an increased risk of all-cause mortality and myocardial infarction only when LTPA was low or moderate [14].

Concerning sex differences, data from the meta-analyses of prospective studies showed that high OPA levels were associated with an increased risk of mortality in men, but not in women (with even a tendency for an inverse association) [12]. Recent data from the Norwegian Cause of Death Registry, with 437,378 participants, showed that moderate to high OPA contributed to longevity in men [15]. However, OPA did not seem to affect longevity in women [15]. These differences can suggest a gender response to OPA or the fact that men are more likely to be involved in physically demanding work than women, causing dissimilar stress on the cardiovascular system [12].

OPA has also been associated with cardiometabolic risk factors. A low OPA has been associated with an increase in hypertensive status in Korean women [16]. However, a protective effect of OPA on diabetes and hypertension was reported in a cohort of 5,157 participants [17]. In contrast, in a Taiwanese cohort of 3,296 workers, a high OPA was associated with a lower risk of abdominal adiposity and elevated triglycerides and diastolic blood pressure (DBP), but with a higher risk of elevated systolic blood pressure (SBP) [18]. Metabolic syndrome (MS) incidence and impaired insulin resistance were significantly related to nonmanual labor, a proxy for low OPA activity, in a cohort of 2,348 middle-aged Korean men [19].

Therefore, PA could have differential health effects depending on whether LTPA or OPA was considered. The possible opposite health effects of LTPA and OPA have been typified as the so-called PA health paradox [20], in which, while PA in the leisure time improves health, OPA could be associated with a positive or negative impact on health. The results of recent cross-sectional analyses in the frame of the Copenhagen City Study highlight the importance of considering the PA health paradox, at least for some risk factors for CVD [20]. In this context, the aim of the present study was to evaluate the association between OPA and cardiometabolic risk factors in adults, both men and women, from 18 to 65 years old. Our hypothesis was that OPA would be independently related to cardiometabolic risk in both men and women.

## 2. Materials and Methods

### 2.1. Design of the Study

The present cross-sectional study was carried out from 1 January to 31 December 2017 at the company Fomento de Construcciones y Contratas (FCC, www.fcc.es, accessed on 15 January 2023), a Spanish building company, S.A. Delegation of Catalonia. The FCC Group handles environmental services, end-to-end water management, infrastructures, cement, and real estate management.

All participants signed a written informed consent form. The study design was approved and agreed upon by the security and health committees of all company worksites and worksite unions. A certificate of ethical approval from the Global Security and Health Committee of the FCC S.A. Delegation, and another from Catalan Public Services, were also obtained. The present cross-sectional study followed the STROBE criteria [21] (Table S1).

### 2.2. Participants and Public Involvement

Participants involved in the approval of the ethics statement were employees of FCC S.A. Delegation, aged 18 to 65, who were active workers during the present cross-sectional study.

### 2.3. Inclusion and Exclusion Criteria

To be eligible for inclusion, participants had to (1) be an active employee (not on sick leave) of the FCC S.A. Delegation with at least one year of service, (2) be ≥18 to 65 years old, and (3) had to have a medical visit in 2017. Exclusion criteria were those who did not fulfill all of the inclusion criteria.

### 2.4. Data Collection

Every year, the company performs an optional medical check-up for all employees and collects data on age, sex, anthropometric measurements, routine laboratory biochemical parameters, lifestyle characteristics, and diagnosed medical conditions. Data from the 2017 visit were used in this study. Measurements and data of employees were collected by the physician and the nurse of the company. They worked together with the whole research team on the data analyses. The medical check-up had a duration of 30–40 min per employee. Data collected were anthropometric and medical conditions, blood sample analyses, and lifestyle characteristics using questionnaires, as specified in the following sections. The objective of check-ups was to contribute to assess the health of the employees and they were not linked to any employment requirement.

### 2.5. Anthropometric Data and Associated Medical Conditions

Anthropometric data were weight (kg) measured with a roman scale (calibrated every year); height (m) measured using a wall-mounted stadiometer (Tanita Leicester Portable; Tanita Corp., Barcelona, Spain); waist circumference (WC) (cm) measured above the iliac crest; and hip circumference (cm) using a 150 cm anthropometric steel measuring tape, following the Lohman manual [22]. Body mass index (BMI) (kg/m^2^) was calculated and categorized using the World Health Organization (WHO) thresholds (BMI ≥25 kg/m^2^ as overweight (OW) and ≥30 kg/m^2^ as obesity). Diagnoses of abdominal obesity (WC ≥102 cm in men and ≥88 cm in women) were assessed [23,24]. The waist−hip ratio was calculated as the ratio between WC and hip circumference. A high waist−hip ratio was considered to be unhealthy when >1 for men and >0.9031 for women [23]. SBP and DBP were collected (mmHg) using the automatic sphygmomanometer OMRON HEM-907; Peroxfarma, Barcelona, Spain), which was calibrated every year following the correct standards. Pulse pressure was calculated as SBP minus DBP. Employees were sitting at rest for approximately 10 min with the arterial pressure monitor on the arm, the physician measured the arterial pressure three times, and the mean of the three measurements, with 1 min interval in between them, was used. Hypertension was defined as an SBP ≥140 mmHg and/or DBP ≥90 mmHg [25] or the use of hypotensive drugs.

### 2.6. Laboratory Data and Associated Medical Conditions

Fasting blood samples were taken with participants in the fasting state through a catheter in the antecubital vein. Blood was collected in Vacutainer tubes with K2EDTA anticoagulant. Blood samples were centrifuged at 1500× *g* for 15 min, and 2.8 mL of plasma was finally recovered. Protease Inhibitor Cocktail (PIC; Sigma-Aldrich, Tres Cantos, Spain) was added to the plasma at a 1/100 (1 μL of PIC for 100 μL of plasma) concentration. All of the samples were stored at −80 °C until processing. Plasma glucose, total cholesterol, and triglyceride values were obtained by standardized routine laboratory methods.

Hypercholesterolemia was defined as a total cholesterol ≥200 mg/dL, hypertriglyceridemia as triglycerides ≥ 150 mg/dL, and dyslipidemia was considered when both cholesterol and triglycerides were higher than these thresholds. DM was reported by the employee and assessed by the family physician.

### 2.7. Lifestyle Data

Tobacco consumption was registered in the clinical history of each employee fulfilled in the check-up visit. Participants were categorized as smokers or nonsmokers according to a specific question concerning whether they smoked or not, and how many cigarettes/day. In addition, alcohol consumption was registered by a question done in the check-up visit, concerning whether they drank alcohol or not, and the quantification of the alcohol and type of alcohol consumed [26]. Participants were categorized as (1) nondrinkers or with (2) low alcohol consumption (<28 standard drink units (SDUs)/week in males and <17 SDUs/week in females), (3) medium alcohol consumption (>28 SDUs/week in males and >17 SDUs/week in females), and (4) high alcohol consumption (>28 SDUs/week in males and >17 SDUs/week in females when participants were taking any kind of medication or if they had a chronic disease). Global PA (including both OPA and LTPA) was recorded by means of the Catalan Physical Activity Questionnaire [27] based on the International Physical Activity Questionnaire [28], and was classified as high, moderate, or low, according to the GPAQ Analysis Guide [28]. OPA was registered according to the Compendium of Physical Activities [29] and work categories of the International Labour Organization [30]. OPA was classified as low when there were ≤3 metabolic equivalents of task (METs) in work hours/day and moderate−high when there were >3 METs in work hours/day, according to the GPAQ questionnaire [31]. In this questionnaire, the questions, referred to last week, were (1) “How many days did you do vigorous physical activity?”, (2) “How many days did you do at least a short period of moderate-intensity physical activity?”, (3) “How many days did you walk at least 10 min?”. Possible answers were “From 1 to 7 days”, and minutes of the most representative day were also asked. In addition, a fourth question, “How many hours did you sit on a non-holiday day? (Choose the most representative day)”, was included.

### 2.8. Employee OPA and Socioeconomic Characteristics

Employees were classified in work categories based on the International Labour Organization [30] (Table S2) and linked with socioeconomic level into one of the following: (a) managers: administrative and commercial managers/production and specialized service managers (high socioeconomic levels: directors and managers); (b) drivers and mobile plant operators (medium socioeconomic level: intermediate occupation); (c) supervisor of operators (medium socioeconomic level: intermediate occupation); (d) cleaners and helpers (low and very low socioeconomic level: primary qualified, half-qualified and non-qualified); and (e) plant and machine operators and assemblers (low and very low socio-economic level: primary qualified, half-qualified, and nonqualified).

### 2.9. Sample Size Calculation

In 2012, the National Health Survey of Spain (ENSE 2011/12), carried out by the Ministry of Health, Social Services, and Equality, in collaboration with the National Institute of Statistics, showed that from the 21,007 adults who answered the survey, 8640 employees were active, and from them, 14.8% presented hypercholesterolemia, 12.7% hypertension, and 3.9% DM, which are three cardiometabolic risk factors.

On this basis, we selected hypercholesterolemia as the most frequent cardiometabolic risk factor in our population. Accepting an alpha risk of 0.05 and a beta risk of 0.2 in a two-sided test, 253 participants in each group would be necessary to detect a difference greater than or equal to 10 mg/dL in the total cholesterol. The standard deviation for total cholesterol in a southern European population has been estimated to be 37 mg/dL [32]. A drop-out rate of 10% was anticipated.

### 2.10. Statistical Analyses

Continuous variables were presented as the mean and standard deviation (SD), and categorical variables were presented as percentages. Shapiro−Wilk test was used for assessing the parametricity of the variables. ANOVA was used to compare continuous variables, and the chi-square test was used to compare the categorical variables. Logistic regression models were used to analyze the associations between OPA and cardiometabolic risk factors regarding categorical (dichotomic) variables, and multivariate linear regression models were used for continuous variables. All of the models were adjusted by age, sex, alcohol consumption, and global PA. All data were analyzed using SPSS V.27.0 for Windows (SPSS Inc., Chicago, IL, USA). The level of statistical significance was set to *p* < 0.05.

## 3. Results

Seven hundred fifty-one employees were included. Most of the population were male (72.7% (*n* = 546/751)). The mean (±SD) age of the population was 45.2 (±9.2) years. A total of 51.7% (*n* = 388) of employees were nonqualified (categorized as very low socioeconomic status employees).

Table 1 shows the characteristics of the population depending on their OPA. Almost half of the employees, 44.5% (*n* = 334), were categorized as having low OPA. The percentage of females in the low OPA group (10.8%, *n* = 36) was significantly lower than that in the high OPA group (40.5%, *n* = 169) (*p* < 0.001). In females, the mean age was higher in the high OPA group than in the low OPA group (*p* = 0.003). Tobacco consumption was higher in males with high OPA than in those with low OPA (*p* = 0.029). In the total population, BMI, WC, waist−hip ratio, cholesterol, and triglycerides were lower (*p* < 0.05) in the high OPA group than in the low OPA group. Cohen’s d and effect size r for differences between low and high OPA groups were: d = 0.211, r = 0.105; d = 0.364, r = 0.179; d = 0.421, r = 0.205; d = 0.264, r = 0.131; d = 0.240, r = 0.119 for BMI, WC, waist−hip ratio, cholesterol, and triglycerides, respectively. When the analyses were performed by sex, BMI, WC, waist−hip ratio, and total cholesterol were significantly lower (*p* < 0.05) in males with high OPA than in those with low OPA. Concerning females, those in the high OPA group had higher levels of SBP and DBP (*p* < 0.05) than those in the low OPA group, reaching a borderline value for pulse pressure (*p* = 0.061).

Concerning medical conditions (Table 2), in the total population, lower rates of overweight plus obesity, DM, hypertriglyceridemia, and dyslipidemia (*p* < 0.05) were observed in the high OPA group compare with the low OPA group. When analyses were performed by sex, males presented a similar pattern to that of the global population.

Table 3 shows the association between OPA and cardiometabolic risk factors adjusted by age, sex, alcohol consumption, and global PA. In both the total and male populations, significant inverse associations were observed between OPA and weight, BMI, WC, waist−hip ratio, and total cholesterol (*p* < 0.05), pointing out an improvement, with lower values, in these parameters at high OPA levels. In contrast, in the female group, only a borderline positive association (*p* = 0.063) between OPA and SBP was observed, suggesting a possible, but not confirmed, increase in SBP when OPA increased.

Table 4 shows the association between OPA and cardiometabolic risk medical conditions. Significant inverse associations, with odds ratios lower than 1, were observed for OPA and dyslipidemia in the total population and in both sexes (*p* < 0.05). Thus, an increase in OPA was associated with a better lipid profile both in the total population and by gender. Similarly, inverse significant associations were also observed between OPA and overweight plus obesity in the total population and in males (*p* < 0.05), pointing out the beneficial effects of OPA avoiding weight gain. In males, a borderline inverse association was obtained between OPA and DM (*p* = 0.051), suggesting a possible, but not confirmed, decrease in DM incidence when OPA increased. In agreement with the data obtained in Table 1, a direct significant association of OPA and tobacco consumption was obtained in males (*p* = 0.048), pointing out higher smoking habits in the high OPA group. No significant associations were observed in females among OPA and cardiometabolic risk medical conditions, other than dyslipidemia.

## 4. Discussion

The results of the present study show that OPA is associated with a better profile of some cardiometabolic risk factors, particularly in males. Although differences in the females could point to a bias according to sex, they are probably related to the low prevalence of females in the low OPA group. As other studies with results in the same line that ours have identified, gender differences could also be attributed to a non-objective OPA measurement; also, men are more likely to be involved in physically demanding work than women, or gender differences in the cardiometabolic risk factors’ response to OPA occur [10,33,34]. High OPA was associated with less weight, BMI, WC, waist−hip ratio, and total cholesterol values in the total and male populations. Consequently, the rate of overweight plus obesity was lower at high OPA in these populations. Males with high OPA had less DM incidence, and in both sexes, dyslipidemia was lower at high OPA values.

In our study, obesity and overweight and their related parameters were consistently reduced with an increase in OPA, both in univariate and multivariate analyses, in both the total and male populations. Our results differ from those obtained in the Chilean National Health Survey 2009–2010 [17], in the sense that in this study (14), OPA was not associated with obesity outcomes. In contrast, our results agree with those obtained in a Taiwanese survey, in which individuals with high OPA had a lower incidence of abdominal adiposity or hypertriglyceridemia [15]. Our results also agree with those obtained in a nurse’s survey in which work posts with low OPA, such as managers or supervisors, were significantly more likely to be overweight or obese than staff nurses [35]. We observed a beneficial effect of OPA on dyslipidemia in both sexes. Our data agree with those reported by the CESCAS I study in 7,512 adults from South American populations, with a better profile of lipid parameters associated with high OPA [36], in which a full adjustment of the models by potential confounding variables, including age, sex, and PA, as in our study, was performed. Contradictory data have been obtained concerning OPA and blood pressure in females. In a cross-sectional study, female cleaners with high OPA showed increased SBP and pulse pressure [37], but opposite data have also been reported [13]. In our study, when raw data were evaluated, both SBP and DBP were higher in high OPA females than in low OPA females. When data were adjusted by age, alcohol consumption, and global PA, however, only a borderline direct association of SBP with OPA remained.

Thus, from our data, an association between high OPA levels and a better profile of several cardiometabolic risk factors exists. However, how do our results fit within the so-called PA health paradox with an increased risk of CVD with high OPA levels? One factor that could explain the discrepancies between the protective effect of OPA on cardiometabolic risk factors, but the contrary when CVD incidence is evaluated, is that high OPA workers have a low socioeconomic status, which is a well-known factor for CVD risk [38]. The fact that in our study we used occupation categories as a proxy for OPA does not allow us to adjust the models for this variable. Low socioeconomic status is linked with high tobacco consumption [39], as is reflected in our study in the relationship between high OPA and tobacco consumption. Anxiety and mood disorders, such as depression, which is also well-known CVD risk factors [40], are more prevalent in lower socioeconomic groups than in higher socioeconomic groups [41]. An increase in inflammatory status, low work control, fatigue, and exhaustion, among others, have been proposed as factors for explaining the CVD risk associated with high OPA [42]. Thus, factors involved in atherogenic risk, other than those examined in this study, could account for explaining the paradox. Another proposed explanation for the PA paradox is the differences in the characteristics of PA. LTPA often includes dynamic movements at conditioning intensity levels sufficient to improve cardiorespiratory fitness over short time periods with enough recovery time. In contrast, work often requires static and other non-conditioning activities over several hours per day without sufficient recovery time [20]. Differences among results in studies assessing the association of OPA and cardiometabolic risk factors could also be attributed to (1) the heterogeneity of the populations involved; (2) differences in the possible confounding variables used in the models; and (3) differences in OPA measurements, from proxies of occupational categories to direct measurement units.

The study has several limitations. First, as a cross-sectional study, it cannot provide cause-effect relationships but only associations. Second, the main variable, the OPA measurement, was collected by questionnaire and not directly measured with an accelerometer. As previously mentioned, we used occupation categories as a proxy for OPA activity, which did not allow us to adjust the model by socioeconomic status. Third, we only had data on global PA (OPA + LTPA) and thus were unable to adjust only for LTPA. Fourth, males represented most of the sample. Therefore, the generalizability of our findings was limited.

From our results, workers with a low OPA must be aware of the risk of sedentarism and be encouraged to compensate with the practice of LTPA and/or active com-muting PA. Data from the 2014–2016 Survey of Health of Wisconsin show that individuals classified as having low OPA levels were less likely to meet the U.S. Federal reported aerobic PA guidelines than individuals who were classified as having high OPA levels [43]. Similarly, nurses with passive jobs were significantly less likely to perform aerobic PA [35]. Interventions addressing modifiable behavioral risk factors for chronic disease would be advisable in low OPA workers. The limited number of interventions made at present, however, did not permit us to draw any conclusion on the proper interventions to be performed or their cost/effectiveness [44]. Further investigation of the association between OPA and health is needed. This issue has been recently reinforced by the WHO guidelines on PA and sedentary behavior [45].

## 5. Conclusions

In our study, OPA was associated with a better profile of cardiometabolic risk factors, particularly in males. The lack of associations obtained in the female group, except in the case of dyslipidemia, could be related to the low sample size of females in the low OPA group. The fact that our models were also adjusted by global PA highlights the associations obtained as being independent of the effects of LTPA. From our results, workers with low OPA activity must be aware of the risk of sedentarism and be encouraged to compensate with the practice of LTPA and/or active commuting PA. In addition, interventions addressing modifiable behavioral risk factors for chronic disease would be advisable in low OPA activity workers. Further investigation of the association between OPA and health is needed.

Future actions for improving cardiometabolic risk factors could consider the results of this cross-sectional study and sex differences.

## Figures and Tables

**Table 1:** Characteristics of the participants according to occupational physical activity (OPA).

	Low OPA			Moderate-High OPA		
Variable	Total (*n* = 334)	Male (*n* = 298)	Female (*n* = 36)	Total (*n* = 417)	Male (*n* = 248)	Female (*n* = 169)
Age, y, mean ± (SD)	45.2 ± 9.2	45.6 ± 9.3	41.6 ± 7.5	45.2 ±10.2	44.2 ± 10.5	46.7 ± 9.5 †
Tobacco consumption, % (*n*)	37.4 (125)	37.6 (112)	36.1 (13)	42.4 (177)	47.2 (177) *	35.5 (60)
Alcohol consumption,% (*n*)	28.7 (96)	29.9 (89)	19.4 (7)	22.5 (94)	29.8 (74)	11.8 (20)
Body mass index, kg/m^2^	28.7 ± 5.1	29.0 ± 5.0	26.5 ± 4.6	27.6 ± 5.3 †	27.2 ± 5.8 †	27.2 ± 5.5
Waist, cm	97.9 ± 13.8	99.5 ± 12.9	85.5 ± 14.5	92.9 ± 13.7 ‡	96.0 ± 13.4 †	88.3 ± 12.9
Waist-hip ratio	0.956 ± 0.1	0.970 ± 0.1	0.951 ± 0.1	0.914 ± 0.1 ‡	0.840 ± 0.1 †	0.860 ± 0.1
Systolic Blood Pressure, mmHg	134 ± 18	136 ± 18	118 ± 12	132 ± 19	135 ± 20.	127 ± 18 †
Diastolic Blood Pressure, mmHg	82 ± 12	83 ± 12	74 ± 10	80 ± 11	81 ± 11	79 ± 12 *
Pulse Pressure, mmHg	13.5 ± 0.7	13.7 ± 0.8	9.1 ± 1.5	14.0 ± 0.7	14.7 ± 0.9	12.1 ± 0.9
Glucose, mg/dL	106 ± 44.3	108 ± 46.1	97 ± 27.1	103 ± 36.2	105 ± 38.0	96 ± 30.3
Cholesterol, mg/dL	201 ± 38.3	203 ± 38.2	183 ± 35.5	191 ± 37.4 *	189 ± 34.8 †	196 ± 43.6
Triglycerides, mg/dL	146 ± 86.3	153 ± 89.2	97 ± 35.7	126 ± 80.4 *	136 ± 87.8	99 ± 48.0

*p* by ANOVA or Chi^2^ tests. OPA: occupational physical activity; SD: standard deviation; Low OPA: ≤3 METs; High OPA: >3 METs. * *p* < 0.05, † *p* < 0.01, ‡ *p* < 0.001 versus low OPA group.

**Table 2:** Medical conditions of participants according to occupational physical activity (OPA).

	Low OPA			Moderate-High OPA		
Variable	Total (*n* = 334)	Male (*n* = 298)	Female (*n* = 36)	Total (*n* = 417)	Male (*n* = 248)	Female (*n* = 169)
Overweight + Obesity, % (*n*)	79.3 (265)	82.2 (245)	55.6 (20)	65.0 (271) ‡	69.0 (171) ‡	59.2 (100)
Obesity, % (*n*)	33.8 (113)	34.9 (104)	25.0 (9)	27.8 (116)	27.0 (67)	29.0 (49)
Abdominal Obesity, % (*n*)	33.5 (112)	33.9 (101)	30.6 (11)	33.3 (139)	26.6 (66)	43.2 (73)
Hypertension, % (*n*)	18.6 (62)	20.1 (60)	5.6 (2)	14.4 (60)	14.1 (35)	14.8 (25)
Diabetes mellitus, % (*n*)	7.5 (25)	8.4 (25)	0 (0)	3.5 (15) *	4 (10)	3 (5)
Impared fasting glucose, % (*n*)	7.5 (25)	8.1 (24)	2.8 (1)	6.7 (28)	10.5 (26)	1.2 (2)
Hypercholesterolemia, % (*n*)	63.2 (115)	64.8 (103)	47.8 (12)	57.6 (106)	53.6 (71)	68.6 (35)
Hypertriglyceridemia, % (*n*)	22.8 (76)	23.5 (70)	16.7 (6)	13.4 (56) *	19 (47)	5.3 (9)
Dyslipidemia, % (*n*)	19.8 (66)	20.5 (61)	13.9 (5)	9.6 (40) ‡	12.5 (31) †	5.4 (9)

*p* by ANOVA or Chi^2^ tests. OPA: occupational physical activity; Low OPA: ≤3 METs; High OPA: >3 METs. * *p* < 0.05, † *p* < 0.01, ‡ *p* < 0.001 versus Low OPA group.

**Table 3:** Association between occupational physical activity (OPA) and cardiometabolic risk factors.

	Total (*n* = 751)		Male (*n* = 546)		Female (*n* = 205)	
Variable	β-Coefficient (95% CI)	*p*-Value	β-Coefficient (95% CI)	*p*-Value	β-Coefficient (95% CI)	*p*-Value
Weight, kg	−4.092 (−6.49; −1.69)	0.001	−4.684 (−7.45; −1.91)	0.001	−1.38 (−6.57; 3.81)	0.600
BMI, kg/m^2^	−0.879 (−1.66; −0.10)	0.028	−1.112 (−1.97; −0.25)	0.011	0.134 (−1.83; 2.10)	0.893
Waist, cm	−2.438 (−4.39; −0.48)	0.015	−3.112 (−5.28; −0.94)	0.005	0.545 (−4.19; 5.28)	0.820
Waist/Hip ratio, cm/cm	−0.012 (−0.02; 0.00)	0.043	−0.016 (−0.03; −0.002)	0.021	0.008 (−0.02; 0.03)	0.556
Glucose, mg/dL	−2.188 (−10.5; 6.08)	0.603	−1.830 (−11.5; 7.82)	0.709	−2.653 (−17.9; 12.6)	0.730
Total cholesterol, mg/dL	−9.125 (−16.7; −1.51)	0.019	−13.70 (−22.0; −5.41)	0.001	7.691 (−10.7; 26.1)	0.407
Triglycerides, mg/dL	−13.54 (−30.5; 3.43)	0.118	−16.42 (−36.8; 4.02)	0.115	−3.958 (−24.6; 16.6)	0.703
SBP, mmHg	1.116 (−1.52; 3.75)	0.407	0.213 (−2.75; 3.18)	0.888	5.840 (−0.33; 12.0)	0.063
DPB, mmHg	−0.589 (−2.33; 1.15)	0.507	−1.462 (−3.38; 0.46)	0.135	3.280 (−0.99; 7.55)	0.131
Pulse Pressure, mmHg	1.670 (−0.30; 3.64)	0.097	1.675 (−0.60; 3.95)	0.149	2.452 (−1.78; 6.68)	0.254

Multiple linear regression models adjusted by age, sex, alcohol consumption, and global physical activity (considering both leisure time and work hours). 95% CI, 95% confidence interval. BMI: body mass index; SBP: systolic blood pressure; DBP: diastolic blood pressure.

**Table 4:** Association between occupational physical activity (OPA) and cardiometabolic risk conditions.

	Total (*n* = 751)		Male (*n* = 546)		Female (*n* = 205)	
Variable	Odds Ratio (95% CI)	*p*-Value	Odds Ratio (95% CI)	*p*-Value	Odds Ratio (95% CI)	*p*-Value
Dyslipidemia	0.519 (0.33; 0.81)	0.004	0.549 (0.34; 0.88)	0.013	0.185 (0.05; 0.72)	0.015
Hypercholesterolemia	0.778 (0.50; 1.20)	0.197	0.626 (0.39; 1.01)	0.056	2.072 (0.72; 5.94)	0.175
Hypertension	0.750 (0.48; 1.16)	0.198	0.658 (0.41; 1.07)	0.089	2.125 (0.46; 9.78)	0.333
Diabetes mellitus	0.541 (0.26; 1.10)	0.090	0.465 (0.22; 1.00)	0.051	----	-----
Tobacco consumption	1.367 (0.99; 1.89)	0.058	1.439 (1.01; 2.05)	0.045	1.274 (0.58; 2.82)	0.550
Obesity	0.736 (0.52; 1.03)	0.078	0.699 (0.48; 1.02)	0.062	0.993 (0.42; 2.35)	0.988
OW + OB	0.572 (0.40; 0.82)	0.002	0.511 (0.33; 0.74)	0.001	0.809 (0.36; 1.80)	0.605
Abdominal Obesity	0.798 (0.57; 1.12)	0.194	0.718 (0.49; 1.05)	0.089	1.265 (0.55; 2.88)	0.576

Logistic binary regression models adjusted by age, gender, alcohol consumption, and global physical activity. 95% CI, 95% confidence interval. OW+ OB, overweight and obesity.

## Data Availability

The technical appendix and raw dataset are available from the following authors upon request: elisabet.llaurado@urv.cat and lucia.tarro@urv.cat.

## Supplementary Materials

The following supporting information can be downloaded at: https://www.mdpi.com/article/10.3390/nu15061421/s1. Table S1: STROBE Statement—Checklist of items that should be included in reports of cross-sectional studies; Table S2: Occupational physical activity (OPA) according occupation.
